# The mediating effect of bullying on parental–peer support matching and NSSI behaviour among adolescents

**DOI:** 10.1186/s12889-024-19309-9

**Published:** 2024-07-03

**Authors:** Huaqiang Liu, Zhensong Lan, Xuefang Huang, Qing Wang, Fafang Deng, Juchao Li

**Affiliations:** 1 School of Law and Public Administration, Yibin University, Yibin, 644000 China; 2 School of Public Administration, Hechi University, No. 42, Longjiang Road, Yizhou, Guangxi 546300 China; 3 School of Teacher Education, Hechi University, Yizhou, 546300 China; 4 College of Humanities, Jiangxi University of Finance and Economics, Nanchang, 330013 China; 5 School of Economics and Management, Beijing University of Technology, Beijing, 100124 China

**Keywords:** Adolescents, Parental support, Peer support matching, NSSI, Bullying, Social connectedness

## Abstract

**Background:**

Being subjected to bullying is a significant risk factor for non-suicidal self-injury (NSSI) among adolescents. Parental support, peer support, and social connectedness play protective roles in mitigating NSSI in this population. However, the precise impact of the combined effects of parental and peer support on bullying and NSSI requires further investigation.

**Methods:**

This study employed the Child and Adolescent Social Support Scale, Delaware Bullying Victimisation Scale, Social Connectedness Scale, and the Ottawa Self-Injury Inventory to survey 1277 Chinese adolescents. Polynomial regression analysis and response surface analysis were applied to examine the mediating role of bullying and social connectedness in the relationship between parental and peer support matching and NSSI.

**Results:**

The results indicate that parental support (*r* = 0.287, *P* < 0.001), peer support (*r* = 0.288, *P* < 0.001), and social connectedness (*r* = 0.401, *P* < 0.001) were protective factors against NSSI in adolescents. Conversely, bullying (*r* = 0.425, *P* < 0.001) acts as a risk factor for NSSI in this population. Adolescents with low parental and peer support experienced more bullying than those with high parental and peer support, while those with low parental but high peer support experienced less bullying than those with high parental but low peer support (R^2 = 0.1371, *P* < 0.001). Social connectedness moderated the effect between bullying and NSSI in this model (β = 0.006, *P* < 0.001).

**Limitations:**

Due to the under-representation of participants and lack of longitudinal data support, the explanatory power of causality between variables was limited. Future studies should include national samples and incorporate longitudinal studies to enhance the generalisability and robustness of the findings.

**Conclusion:**

This study reveals the influence mechanism of parental and peer support matching experienced by adolescents on bullying and NSSI and the moderating role of social connectedness. These findings enrich the developmental theory of adolescent NSSI and provide reference for the prevention and intervention of adolescent NSSI behaviour.

## Introduction

Adolescent non-suicidal self-injury (NSSI) is a serious psychological and behavioural problem. Long-term NSSI behaviour can damage individual health and well-being [1], and may ultimately lead to adolescent suicide [2–5]. The reported prevalence of NSSI among Chinese adolescents ranges from 14 to 34% [6–9], indicating the severity of the issue. Bullying has a significant negative impact on the physical and psychological well-being of adolescents, serving as one of the predictive factors for NSSI behaviours among adolescents [10]. Studies indicate that receiving social support acts as a preventive measure against NSSI behaviours [11–13]. As a subsystem of the social support system, parental support contributes to the prevention of NSSI behaviour among adolescents [14]. Similarly, peer support, another important subsystem of the social support system, influences adolescents’ NSSI behaviours [15]. In summary, the experience of bullying among adolescents is closely associated with the occurrence of NSSI behaviours, and positive parental and peer support play a crucial role in preventing NSSI behaviours among adolescents. Nonetheless, the precise mechanisms by which parental and peer support influence adolescent NSSI behaviours among bullying victims remain unclear, warranting a more comprehensive exploration.

This study delves into the combined effects of parental and peer support on adolescent bullying and NSSI and examines how social connectedness mediates the relationship between adolescent bullying and NSSI. The goal of this study is to establish a theoretical foundation for strategies aimed at preventing and intervening in adolescent NSSI.

### Relationship between bullying and NSSI

Bullying is defined as ‘deliberate, repetitive, negative (unpleasant or hurtful) behaviour by one or more individuals against a person who has difficulty defending himself or herself’ [16]. According to general strain theory (GST), bullying can have long-term negative physical and psychological effects on adolescents [10]. Previous studies have shown that bullying is associated with NSSI risk in adolescents [10, 17] and is an important predictor [18]. Moreover, adolescents who have experienced bullying are at a much higher risk of developing NSSI than those who have not [10]. Bullied adolescents may use NSSI to seek attention and help [19], relieve bullying-related stress [20], and reduce their inner guilt and pain by punishing themselves [3, 21, 22]. For instance, when individuals face interpersonal pressures such as physical or verbal abuse, they may find it challenging to effectively handle the ensuing distress [20].

Consequently, they may perceive NSSI as a coping strategy to regulate and alleviate acute negative effects or emotional arousal [20], aiming to alleviate their suffering [21]. Therefore, preventing or reducing bullying among adolescents can directly or potentially decrease the frequency of their NSSI behaviours and reduce negative harm [10, 11, 17].

### Relationship between parental and peer support and NSSI

Drawing on Bronfenbrenner’s ecological systems theory [23], individual development is understood as a product of reciprocal interactions between individuals and their surrounding environments. Within this framework, peers and parents have emerged as pivotal influencers of adolescents’ developmental environment [24].

The functional theory of NSSI [25] posits that individuals experiencing subordinate status or social failure may resort to NSSI behaviours to elicit attention and support. For instance, inadequate family support and peer pressures can influence individuals’ engagement in NSSI behaviours [4, 26–28]. Research has shown that positive family functioning and support serve as effective deterrents for adolescent NSSI behaviours [1, 14, 29] and may facilitate their cessation [30]. Conversely, detrimental family relationships may exacerbate stress among adolescents, consequently leading to NSSI behaviours [31]. Peers also significantly influence adolescent growth and development, and enhancing positive peer relationships and providing peer support may mitigate interpersonal issues, alleviating NSSI behaviours [11].

Furthermore, positive family relationships overall may moderate interpersonal issues (e.g. peer relationships) and contribute to mitigating or reducing NSSI behaviours [11, 12]. Negative occurrences in peer relationships can precipitate adolescent NSSI behaviours [15], whereas adolescents with close parent-child relationships may exhibit lower NSSI behaviours and serve as protective factors for those demonstrating NSSI behaviours due to poor peer relationships [14]. Hence, parental and peer support play a critical role in preventing adolescent NSSI. However, elucidating how parental and peer support collectively influence adolescent NSSI behaviours warrants further investigation.

### Relationships among parental support, peer support, bullying, and NSSI

The environmental theory model of NSSI suggests that an individual’s living environment is a stable system, and when the equilibrium of the system is threatened, individuals may engage in NSSI to restore the physical balance caused by environmental factors [32]. One study demonstrated that while parental support can serve as a protective factor against bullying and other forms of harm, it may also increase the risk of engaging in NSSI behaviour [27]. This is because adolescents may adopt and even repeat NSSI behaviours to receive parental attention [33].

The life model theory argues that life problems reflect an imbalance between individuals and the environment and that poor interpersonal processes affect the outcome of an individual’s personal life and their response to environmental factors [34]. Some studies have suggested that adolescent NSSI behaviour usually occurs in social environments with negative interpersonal influences and emotional distress [17, 35]. For adolescents, bullying and being bullied are negative interpersonal events [36] and may lead to NSSI without intervention or adjustment, thereby affecting their interpersonal communication and emotions [10, 28]. Individuals who experience bullying are gradually socially excluded, resulting in lower social competence and self-esteem [37], and a lack of peer support can increase loneliness, social frustration, and negative self-worth [38].

In summary, parental support acts as a buffer against the harm caused by bullying and NSSI among adolescents. Although adolescent interpersonal relationships are interrelated with NSSI, the relationship between peer relationships, bullying, and NSSI behaviours remains unclear. Therefore, further exploration is needed to understand how parental and peer support jointly influence the relationship between bullying and NSSI behaviours among adolescents.

### Role of social connectedness in bullying and NSSI

Social connectedness refers to the subjective perception of intimacy in one’s surrounding interpersonal relationships and the regular cognition of interpersonal patterns, reflecting one’s internal sense of belonging [39, 40]. One study showed that social connectedness is a protective factor against common psychological distress symptoms and is negatively correlated with shyness, anxiety, loneliness, and interpersonal problems [41]. When bullied adolescents lack social connectedness, their incidence of NSSI behaviours may increase [42, 43]. Social connectedness can moderate the relationship between risk factors and suicide [44].

For adolescents, social connectedness reflects the ability to effectively perceive and utilise social support from parents, peers, and others. Bullying victims’ perceived social support can alleviate their internal distress [37]. In contrast, a lack of perceived social support [45] is closely related to NSSI behaviour [46]. For instance, in cases where parents struggle to find effective approaches to engage with their children [1], children with NSSI behaviour may choose to seek outside help rather than from their parents [47, 48]. Hence, promoting enhanced social connectedness may have a beneficial effect on the prevention of NSSI among adolescents [49]. Therefore, this study explored the moderating role of social connectedness between parental and peer support in the relationship between bullying and NSSI.

### Current study

NSSI among adolescents is a serious psychological and behavioural issue, with long-term NSSI behaviours harming individuals’ health and well-being [1]. Currently, Chinese adolescents face serious problems with NSSI. Bullying is an important predictive factor of NSSI among adolescents [10], while positive parental and peer support play important preventative roles [11–13]. Although the importance of parental and peer support in reducing NSSI among adolescents has been recognised, the mechanisms of these factors among adolescents who have experienced bullying are still unclear.

Furthermore, social connectedness may help alleviate the impact of bullying and NSSI among adolescents [39, 40]. However, it is still unclear which factor, parental or peer support, plays a larger role when adolescents experience bullying at school, or how the interaction effect of both these factors influences the mechanisms of bullying and NSSI among adolescents.

**Fig. 1:**
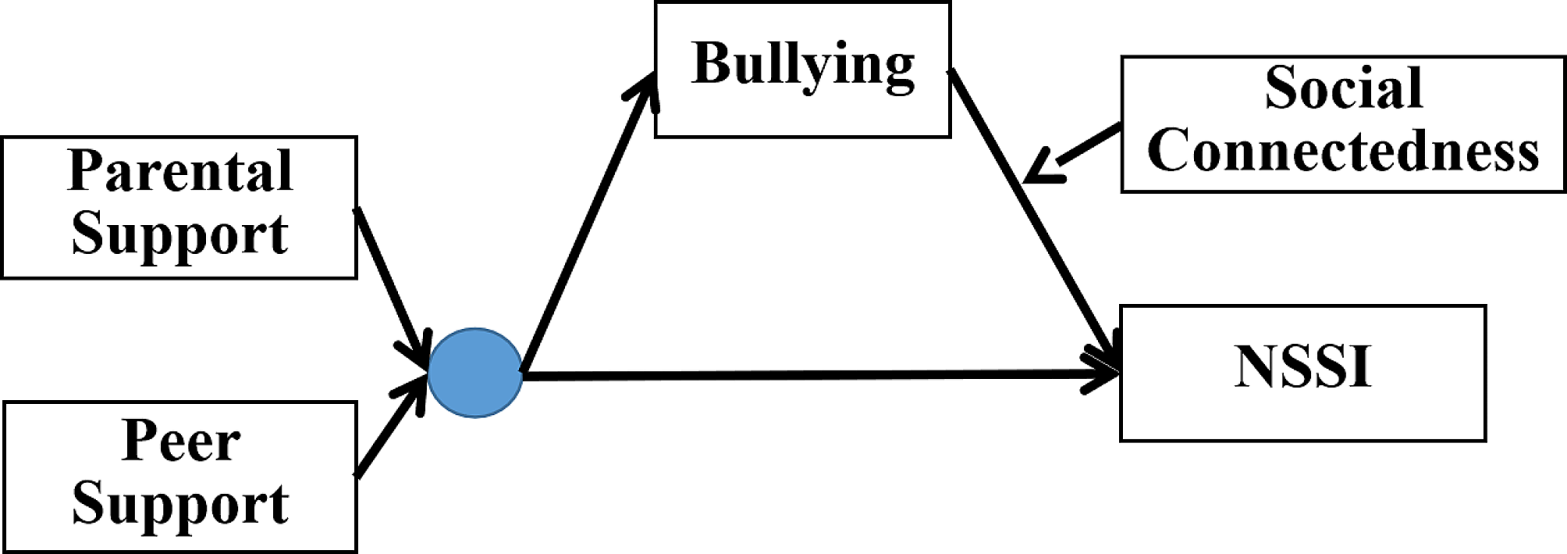
Hypothesized theoretical model

Therefore, this study proposes a moderated mediation model (see Fig. 1) to explore the mechanisms of parental and peer support in relation to bullying and NSSI among adolescents, and to investigate the mediating role of social connectedness between bullying and NSSI. This study provides a theoretical basis for the prevention and intervention of NSSI in adolescents and is based on the following research hypotheses:

#### H1

Bullying among adolescents is significantly, positively correlated with NSSI.

#### H2

High levels of parental and peer support have a significant, positive effect on NSSI among adolescents.

#### H3

Bullying mediates the interaction effect of parental and peer support on NSSI.

#### H4

Social connectedness plays a moderating role in parental-peer support matching on bullying and NSSI.

## Research methods

### Participants

The participants were middle- and high-school students from cities L and Y in Sichuan Province, China. Using a cluster sampling method at the class level, adolescents from 24 classes were selected from four schools in the two cities. The selected schools comprised two junior and two senior high schools, with two classes chosen from each grade level. Cities L and Y in Sichuan Province have medium development levels, and the sample from the four schools includes both urban and rural areas, demonstrating good representativeness. A questionnaire survey was conducted using offline responses. Prior to the survey, informed consent was obtained from all participants along with their parents and schools. Detailed information regarding the study’s purpose, content, and utilisation was also provided. In total, 1320 questionnaires were distributed, and 1277 valid samples were retained after eliminating invalid samples, accounting for 96.74%.

The average participant age was 14.34 years (SD = 1.72), with 678 boys (53.1%) and 599 girls (46.9%). Regarding grade distribution,211 were in seventh grade (16.5%), 245 in eighth grade (19.2%), 255 in ninth grade (20.0%), 194 in tenth grade (15.2%), 183 in eleventh grade (14.3%), and 189 in twelfth grade (14.8%). The sample included 269 (21.1%) urban households, 1008 (78.9%) rural households, 193 (15.1%) only children, 1084 (84.9%) non-only children, 603 (47.2%) left-behind children, and 674 (52.8%) non-left-behind children.

### Measurements

#### A. Child and adolescent social support scale (CASSS)

In this study, the revised Chinese version of the CASSS developed by Luo et al. [50] was utilised, and the reliability and validity of this scale were good. It comprises five subscales and 60 items. The Parental Support Scale and Peer Support Scale are subscales of the CASSS, consisting of 12 items each, encompassing emotional, informational, and tangible support (e.g., ‘my parents are proud of me’, ‘my friends understand my feelings’). Participants rated the frequency of each item on a five-point Likert scale (0 = never, 1 = occasionally, 2 = sometimes, 3 = often, and 4 = always). After reverse-scoring transformation, higher scores indicated lower levels of social support.

#### B. Delaware bullying victimisation scale-student (DBVS-S)

The DBVS-S, revised by Xie et al. [51], was used in this study, and the reliability and validity of this scale were good. It comprises 12 items across three dimensions: verbal, physical, and social/relational bullying (e.g., ‘I was teased by other people, and they said some very hurtful things’). Responses were provided on a five-point Likert scale (0 = never, 1 = occasionally, 2 = sometimes, 3 = often, and 4 = always). After the reverse-scoring transformation, the higher the score, the higher the degree of bullying.

#### C. Social connectedness scale (SCS)

The SCS, revised by Fan et al. [52], was used to measure the degree of social connectedness, and the reliability and validity of this scale were good. It comprises 20 items across three dimensions: sense of integration, sense of acceptance, and life involvement (e.g. ‘I feel comfortable in front of strangers’, and ‘I feel isolated from the world around me’). Participants rated each item on a five-point Likert scale (0 = never, 1 = occasionally, 2 = sometimes, 3 = often, and 4 = always). After the reverse-scoring transformation, the higher the score, the lower the social connectedness.

#### D. Ottawa self-injury inventory (OSI)

In this study, adolescent NSSI behaviour was measured according to items on body parts and frequency using the revised OSI (Chinese version) by Zhang et al. [53], comprising 13 items (e.g. ‘deliberately pinching oneself’), and the reliability and validity of this scale were good. Participants responded on a five-point Likert scale (0 = never, 1 = occasionally, 2 = sometimes, 3 = often, and 4 = always). After the reverse-scoring transformation, the higher the score, the more severe the NSSI behaviour.

### Analysis method

SPSS (version 23.0) was used to test the reliability and validity of the research tools and conduct descriptive statistical analyses, correlation analyses, and mediation moderation effect tests on the research variables. This was done to examine the correlations and the mediating and moderating effects of parental support, peer support, bullying, social connectedness, and NSSI. The R programming language was used for polynomial regression and response surface analysis [54]. Estimates and significance tests were conducted on important characteristic data of the three-dimensional response surface, along with the creation of a three-dimensional response surface plot to verify the relationship between the parent-peer support match, bullying, and NSSI.

Previous studies have indicated that polynomial regression and response surface analysis can overcome the limitations of conventional difference score methods and profile similarity index, which may yield falsely inflated results in relevant (inconsistent) studies [9, 55]. The three-dimensional atlas presented by the analysis results depicts the effects of different matching relationships between two variables on the dependent variable, rendering more intuitive conclusions [56]. The formula for the model constructed in this study [57] is as follows:

$$\eqalign{{\rm{QL = }} & {\rm{b0 + b1(PaS) + b2(PeS) + b3(PaS}}{{\rm{)}}^{\rm{2}}} \cr & {\rm{ + b4(PaS) \times (PeS) + b5(PeS}}{{\rm{)}}^{\rm{2}}}{\rm{ + sex}} \cr & {\rm{ + age + e,}} \cr}$$

where PaS represents parental support, PeS represents peer support, and (PaS) × (PeS) represents the cross-term between parental and peer support. The variables b0 to b5 represent the coefficients: b0 is the intercept, b1 is the coefficient of PaS, b2 is the coefficient of PeS, b3 is the coefficient of PaS squared, b4 is the cross-term coefficient, b5 is the PeS squared coefficient, and e represents the error term. Sex and age were included as control variables.

The PaS and PeS measurement indicators were first processed using scale centralisation. Each item was then regressed, and the results were presented in three-dimensional graphs. The impact was mainly judged based on the outcome variables by calculating the values of the slope (a1 = b1 + b2) and curvature (a2 = b3 + b4 + b5) of the PaS = PeS matching curve, the slope (a3 = b1 − b2) and curvature (a4 = b3 − b4 + b5) of the PaS = − PeS mismatching curve, and their significance.

To test the moderated mediation effect, the polynomial regression coefficients mentioned above were used to construct the block variable [58], which is the consistent block variable between parental and peer support. The mediating effect was then tested using the block variables as independent variables.

Finally, following Wen and Ye’s method of testing the mediating effect of moderation [59], this study examined the influence of block variables (PaS-PeS) on adolescent NSSI behaviour, the mediating effect of being bullied (B), and the moderating effect of social connectedness (SC) on this mediating effect. Therefore, three equations were constructed: Eq. (1) estimates the prediction of the independent variable block variable on the dependent variable NSSI; Eq. (2) estimates the block variable’s prediction of the mediating variable bullying; and Eq. (3) estimates the moderating effect of the social connectedness variable on the association between bullying experiences and NSSI, along with the block variable residual effect test on NSSI, with all continuous variables standardised.

## Results

### Common method bias test

Since the data in this study were all based on self-reports from participants, the common method bias is unavoidable. Thus, Harman’s single-factor method was employed to assess the common method bias in the data. The basic assumption of this method is that if a substantial amount of method variance exists, either a single factor will emerge from the factor analysis or one general factor will account for most of the variance. However, the limitations of this method are its inability to control for method effects and its insensitivity to bias detection.

All items were examined using exploratory factor analysis (EFA), and if the variance explained by the first factor was less than a certain critical standard (e.g. 40%), it was considered to not contain any serious common method bias. The analysis results showed that the eigenvalues of the 12 factors were greater than 1, and the first factor explained 27.69% of the variance, which is less than the critical index of 40%. These results indicate that this study did not have a serious common method bias problem.

### Descriptive statistical and correlation analyses

Table 1 provides the average values, standard deviations (SDs), and correlation matrices of each variable. A significant negative correlation was found between gender and bullying (*R* = -0.065, *P* < 0.05), but not between gender and NSSI (*R* = 0.046, *P* > 0.05). Age was negatively correlated with bullying (*R* = -0.110, *P* < 0.001) and NSSI (*R* = -0.160, *P* < 0.001). Parental support, peer support, bullying, social connectedness, and NSSI scores were positively correlated with each other (Rs = 0.287–0.425, Ps < 0.001). Therefore, H1 is supported. The detection rate of at least one NSSI behaviour in this study was 38.4%.

**Table 1:** Descriptive statistics and correlation analysis (*N* = 1277)

Variables	*M*	*SD*	1	2	3	4	5	6	7
1. Sex	0.47	0.50	1						
2. Age	14.34	1.72	0.078**	1					
3. PaS	21.94	12.55	0.045	0.084**	1				
4. PeS	18.53	11.33	-0.103***	-0.073**	0.538***	1			
5. B	6.40	8.19	-0.065*	-0.110***	0.319***	0.352***	1		
6. SC	29.60	14.26	-0.012	-0.004	0.524***	0.625***	0.467***	1	
7. NSSI	3.16	6.92	0.046	-0.160***	0.287***	0.288***	0.425***	0.401***	1

*Note* Statistically significant values: *p < 0.05, **p < 0.01, and ***p < 0.001; sex:0 for boys and 1 for girls; PaS, parental support; PeS peer support; B, bullying; SC, social connectedness; NSSI, non-suicidal self-injury

A response surface analysis was employed to test the effect of the PaS-PeS match on bullying and NSSI [58]. Before the analysis, the proportion of sample responses was analysed to determine whether polynomial regression and response surface analysis were suitable. The results indicated that among the participants, 49.18% (628) had the same level of parental and peer support, 24.90% (318) had more parental than peer support, and 25.92% (331) had less parental than peer support. These proportions met the requirements (> 10% for each category).

### Effects of parental–peer support matching on adolescent bullying exposure

Polynomial regression and response surface analysis techniques were employed to investigate the impact of parental–peer support matching on adolescent bullying (Table 2).

**Table 2:** Polynomial regression results and response surface analysis (*N* = 1277)

Variable	B	B
b0	9.986***	13.389***
Sex	-0.760	-0.847*
Age	-0.569***	-0.523***
b1	0.464***	0.432***
b2	0.518***	0.525***
b3		0.038*
b4		-0.06**
b5		0.079**
a1 = b1 + b2		0.957***
a2 = b3 + b4 + b5		0.056**
a3 = b1-b2		-0.093
a4 = b3-b4 + b5		0.177***
a5 = b3-b5		-0.041
R^2	0.1246	0.1371
ΔR^2	0.1218	0.1323
F-statistic	45.26***	28.8017***

*Notes* Statistically significant values * *p* < 0.05, ** *p* < 0.01, and *** *p* < 0.001; sex:0 for boy and 1 for girl; PaS parental support; PeS, peer support; B, bullying; SC, social connectedness; NSSI, non-suicidal self-injury. The regression coefficients in the table are non-standardised; ΔR^2 represents the change value of the model explanation rate after adding the quadratic terms of PaS^2, PaS × PeS and PeS^2, and R^2 represents the variance explanation rate of the polynomial regression total model; a1 and a2 denote the slope and curvature of the uniform line, a3 and a4 denote the slope and curvature of the non-uniform line, and a5 denotes whether the first principal axis of the curved surface is located on the uniform line. The same below

After controlling for sex and age, bullying levels were positively predicted by parental support and negatively predicted by peer support (*P* < 0.001). The significance of the ΔR^2 for the polynomial model increased (*P* < 0.001), indicating a significant correlation between the change in the independent variables in the opposite direction (i.e., ‘one high and one low’) and the dependent variable. The lateral shift in the response surface along the discordance line was not significant (a5 = − 0.041, *P* > 0.05), indicating that the first principal axis was completely located on the concordance line.

**Fig. 2:**
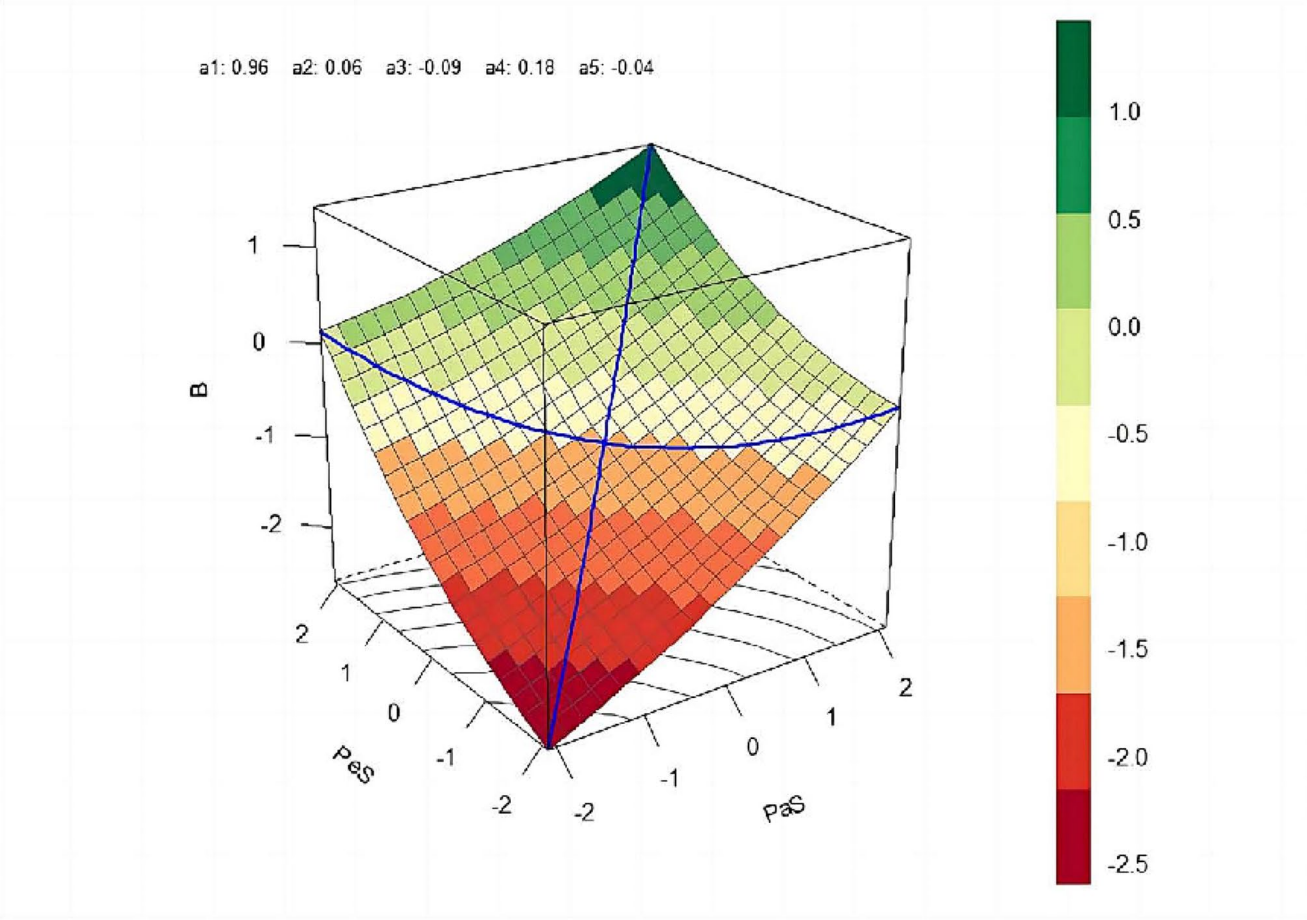
Response surface analysis of parent support (PaS)–peer support (PeS) matching with bullying (B) and NSSI

The slope (a1 = 0.957, *P* < 0.001) and curvature (a2 = 0.056, *P* < 0.01) of the response surface along the line of agreement (PaS = PeS) indicate that the dependent variable is completely consistent with the independent variable as a ‘concave’ rising surface (Fig. 2). The response surface descends along the conformity curve, reaching its lowest position near the stagnation point, and then ascends. The Z-hat value (i.e., difference between concordance lines Z1 and Z2) was calculated using two points selected along the concordance line, plus or minus one SD, showing that when the ‘high-high’ and ‘low-low’ levels of the independent variable are consistent, the dependent variable level is higher (Z-hat = 7.174, 95% CI [6.068, 8.213]). As scores increased, the level of support decreased, implying that adolescents with low parental and peer support experienced higher levels of bullying than those with high parental and peer support, supporting Hypothesis H2.

The curvature (a4 = 0.177, *P* < 0.001) of the response surface along the discordance line (PaS = -PeS) indicates a “concave” incremental change, suggesting that consistency between the independent variables has a negative effect on the outcome variable (Fig. 2). The slope of the discordance line (a3 = -0.093, *P* > 0.05) indicates that the difference in the independent variables is not significantly different from the dependent variable level. However, the Z-hat value along the discordance line shows that the dependent variable level was lower for “high PaS-low PeS” than for “low PaS-high PeS” (Z-hat = -2.231, 95% CI [-4.562, -0.051]). As scores increased, the level of support decreased. This indicates that adolescents with low parental support and high peer support experienced less bullying than those with high parental but low peer support.

### Moderated mediating effect test

Next, we examined the impact of the block variable (FM-HY) on adolescent NSSI, as well as the roles of bullying (QF) and social connectedness (SL) in this relationship. Polynomial regression and response surface analysis included sex and age and were therefore included in the equation as control variables. All variables were normalised (Table 3).

**Table 3:** Moderated mediation model analysis (*N* = 1277)

Variable	Model 1	Model 2	Model 3
	B	NSSI	NSSI
Constant	9.866***	9.003***	5.593***
Sex	-0.703	0.991***	1.080***
Age	-0.504***	-0.663***	-0.518***
V-block	0.652***	0.511***	0.152***
B			0.019
SC			0.069***
B*SC			0.006***
R^2	0.133	0.130	0.270
ΔR^2	0.131	0.128	0.267
F-statistic	64.97***	63.29***	78.41***

*Notes* Statistically significant values * *p* < 0.05, ** *p* < 0.01, and *** *p* < 0.001; sex:0 for boys and 1 for girls; PaS, parental support, PeS peer support; B, bullying; SC, social connectedness; NSSI, non-suicidal self-injury

Model 1 included sex and age as control variables, the V-block (block variable (PaS-PeS)) as the independent variable, and bullying as the dependent variable. Sex had no significant effect on bullying (β = -0.703, *P* > 0.05), while both age (β = -0.504, *P* < 0.001) and the V-block were significant predictors of adolescent bullying (β = 0.652, *P* < 0.001).

Model 2 included sex and age as control variables, V-block as the independent variable, and NSSI as the dependent variable. Sex (β = 0.991, *P* < 0.001) and age (β = -0.663, *P* < 0.001) significantly predicted NSSI levels. The V-block was also a significant predictor of NSSI (β = 0.511, *P* < 0.001).

Model 3 included sex and age as control variables, the V-block as the independent variable, bullying (B) as the mediator, social connectedness (SC) as the moderator, and NSSI as the dependent variable. Sex (β = 1.080, *P* < 0.001) and age (β = -0.518, *P* < 0.001) were significant predictors of NSSI. Both the block variable (V-block) (β = 0.152, *P* < 0.001) and social connectedness (β = 0.069, *P* < 0.001) had significant effects on NSSI, but bullying did not (β = 0.019, *P* > 0.05).

The interaction between bullying and social connectedness had a significant effect on NSSI (β = 0.006, *P* < 0.001). This suggests that the block variables (PaS-PeS), bullying, social connectedness, and NSSI constitute a moderated mediation model, with bullying partially mediating and social connectedness moderating the second half of the mediation path (i.e. the effect of bullying on NSSI). Therefore, H3 and H4 were supported. Table 4 shows the mediating effect of social connectedness at the mean and plus or minus one SD. The results show that in each regression model, the proportion of the mediating effect gradually increased with each increase in social connectedness.

**Table 4:** Mediating effect analysis of bullying at different levels of social connectedness (*N* = 1277)

NSSI	Intermediate effect value	Bootstrap SE	95% CI	Direct effect value	95% CI	Mediating effect(%)
M-1 SD	0.0686	0.0416	[-0.013,0.150]	0.1521	[0.056,0.248]	31.08
M	0.1211	0.0274	[0.067,0.175]			44.33
M + 1 SD	0.1736	0.0343	[0.106,0.241]			53.30

**Fig. 3:**
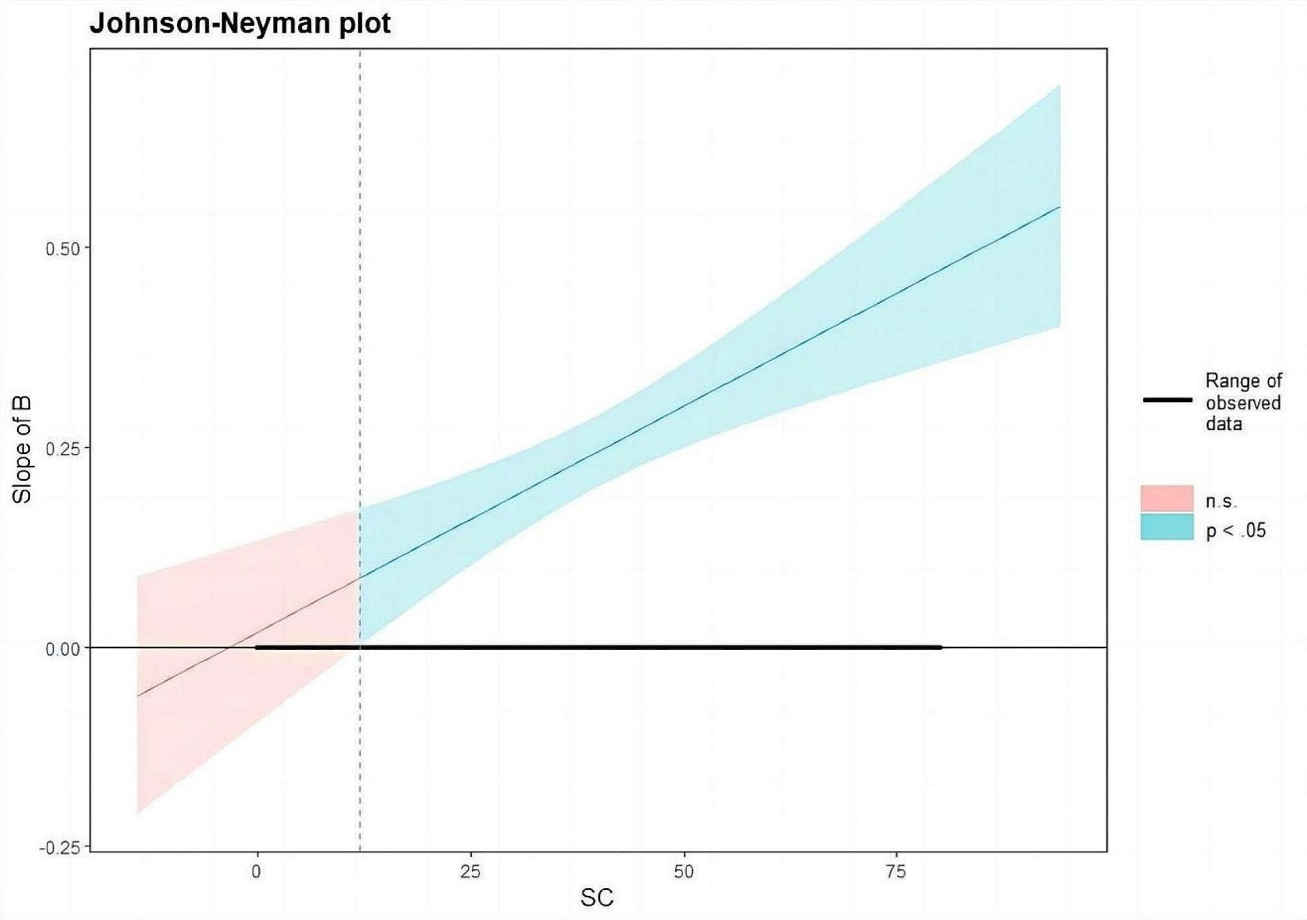
A simple slope graph of the moderating effect of social connectedness (SC) on the effect of bullying (B) on adolescent NSSI

To gain deeper insight into the moderating effect, a simple slope test was used to evaluate the influence of social connectedness on the relationship between bullying and NSSI (Fig. 3). The Johnson-Neyman moderation effect diagram, as suggested by Hayes and Matthes [60], explains the moderating effect between independent and dependent variables. The Johnson-Neyman diagram shows that as social connectedness increases, the slope of bullying on NSSI gradually increases. When social connectedness reached a positive value of 11.93 SD, the predictive effect of bullying on NSSI was not significant. These findings indicate that social connectedness is a protective factor for adolescent NSSI and that high levels of social connectedness can buffer the effects of bullying on NSSI.

## Discussion

Adolescence is a period marked by physical, cognitive, social, and emotional development [61], and can also be a time when NSSI behaviour begins and peaks [25]. In this study, 38.40% of the participants engaged in NSSI behaviour, which is slightly higher than the rates reported in previous studies [6–9]. Drawing on GST and ecosystem theory, this study utilised polynomial regression and response surface analysis to investigate the effects of parental and peer support on adolescent NSSI. Additionally, this study analysed the mediating and moderating effects of bullying and social connectedness.

### Mediating role of bullying

The ecological systems theory [23] underscores the role of the environment in individual growth and development; a supportive family environment contributes significantly to adolescents’ overall healthy development [24]. Family support can effectively prevent [1] or halt [30] adolescent NSSI behaviours. Similarly, enhancing peer relationships and providing peer support can moderate interpersonal problems and mitigate NSSI behaviour [11]. Conversely, negative parent–child relationships can lead to increased problem behaviours and negative emotional experiences in children [62]. Negative events in peer relationships also contribute to adolescent NSSI behaviour [15], while close parent–child relationships can reduce NSSI behaviour and serve as a protective factor for adolescents who develop NSSI due to poor peer relationships [14]. Parental and peer support are crucial factors in the environmental system and are included in this study on the impact of bullying on adolescent NSSI. The results not only show that the ‘parental and peer support’ block variable can directly predict adolescent NSSI but also that bullying plays a mediating role between them.

Previous studies have predominantly explored the influence of parent–child and peer relationships on adolescent bullying and NSSI from the perspective of either parents or peers [1, 10, 15, 33]. However, there are differences in the effects of matching consistency and inconsistency between parents and peers on adolescent psychological well-being [63].

Using polynomial regression and response surface analysis, this study innovatively investigated the influence of parent-peer support matching. The findings revealed that adolescents who lacked adequate parental and peer support experienced a higher incidence of bullying than those who enjoyed strong parental and peer support. Moreover, adolescents with low parental and high peer support experienced less bullying than those with high parental and low peer support. This demonstrates that both parental and peer support are crucial to addressing adolescent bullying and promoting physical and mental health development, with peer support having a more positive impact than parental support on bullying. Real-world examples support this conclusion. For instance, when adolescents face psychological problems or serious crises, although parents are an important source of support [27, 64], they tend to seek more support from peers when their parents are unable to provide emotional support or satisfaction [47, 48]. Additionally, parental preference is significantly higher, and adolescents may engage in NSSI behavior to gain their parents’ attention [33].

### Moderating role of social connectedness

Life model theory posits that life problems arise from an imbalance between individuals and their environment, with poor interpersonal processes affecting personal outcomes and responses to environmental factors [34]. Social connectedness refers to an individual’s subjective perception of intimacy in interpersonal relationships [39, 40], which is closely linked to the quality of interpersonal relationships. Perceived closeness in interpersonal relationships can influence one’s subjective sense of self [39, 40]. Research indicates that social connectedness serves as a protective factor against common psychological distress symptoms and is negatively correlated with shyness, anxiety, loneliness, and interpersonal problems, but positively correlated with self-confidence and social support [41]. Adolescent NSSI behaviours often occur in environments characterised by negative interpersonal influences and emotional distress [17, 35].

Therefore, this study incorporated social connectedness to explore its impact on a mediation model involving block variables (parental and peer support) between bullying and NSSI. The results indicated that social connectedness moderated the second half of the mediation model (i.e. the impact of bullying on NSSI). Social connectedness acted as a protective factor against NSSI, and the influence of bullying diminished as social connectedness increased. This further underscores the role of social connectedness in regulating the relationship between risk factors and behaviours [44]. Perceived social support, including positive parental and peer support, can alleviate the internal distress experienced by bullying victims [37]. Therefore, enhancing social connections and seeking external support are crucial in preventing adolescent NSSI behaviour [49].

## Conclusions

This study revealed that Higher levels of parental support, peer support, and social connectedness were associated with lower rates of NSSI, whereas higher levels of bullying were associated with higher rates of NSSI. Specifically, adolescents with low parental and peer support experienced more bullying than those with high levels of both, while those with low parental and high peer support experienced less bullying than those with high parental and low peer support. Social connectedness moderated the second half of the mediation model, acting as a protective factor against adolescent NSSI, with the impact of bullying on NSSI diminishing as social connectedness increased.

Using polynomial regression and response surface analysis, this study explored the combined effects of parental and peer support on adolescents, revealing that bullying and social connectedness mediated and moderated these effects. These findings contribute to the developmental theory of adolescent NSSI and offer insights for prevention and intervention. However, some limitations constrain the findings. As this is a cross-sectional study, causal relationships cannot be established, particularly regarding the moderating effect of social connectedness, and experimental data support is lacking. Additionally, the sample lacked diversity, consisting of only students from a specific region in China, potentially limiting the generalisability of the findings. Future research should consider using larger and more diverse samples as well as longitudinal data to better understand the development of mental health in adolescents.

## Data Availability

The data used to support the findings of this study are available from the corresponding author upon request.
